# Effect of the Combination of Phosphate-Solubilizing Bacteria with Orange Residue-Based Activator on the Phytoremediation of Cadmium by Ryegrass

**DOI:** 10.3390/plants12142727

**Published:** 2023-07-22

**Authors:** Xin Peng, Rule Zhao, Yuan Yang, Yaoyu Zhou, Yichun Zhu, Pufeng Qin, Mi Wang, Hongli Huang

**Affiliations:** 1 Hunan International Scientific and Technological Cooperation Base of Agricultural Typical Pollution Remediation and Wetland Protection, College of Environment and Ecology, Hunan Agricultural University, Changsha 410128, China; px15773954421@163.com (X.P.);; 2 Chinalco Environmental Protection and Ecological Technology (Hunan) Co., Ltd., Changsha 410021, China

**Keywords:** soil remediation, activator, toxic metal, phosphate-solubilizing microorganism, microbial community structure

## Abstract

Amendments with activators or microorganisms to enhance phytoremediation in toxic-metal-polluted soils have been widely studied. In this research, the production of indoleacetic acid, siderophore, and 1-aminocyclopropane-1-carboxylate (ACC) deaminase by phosphate-solubilizing bacteria was investigated during a pure culture experiment. Pot experiments were performed using Cd-polluted soil with the following treatments: control (CK, only ultrapure water), orange-peel-based activator (OG), and a combination of phosphate-solubilizing bacteria (*Acinetobacter pitti*) and OG (APOG). Ryegrass plant height and fresh weight, Cd content in ryegrass, total and available Cd soil content, soil enzyme activity, and soil bacterial diversity were determined in this work. The findings showed that the height of ryegrass in OG and APOG increased by 14.78% and 21.23%. In the APOG group, a decreased ratio of Cd was 3.37 times that of CK, and the bioconcentration factor was 1.28 times that of CK. The neutral phosphatase activity of APOG was 1.33 times that of CK and catalase activity was 1.95 times that of CK. The activity of urease was increased by 35.48%. APOG increased the abundance of beneficial bacteria and *Proteobacteria* was the dominant bacterium, accounting for 57.38% in APOG. Redundancy analysis (RDA) showed that nutrient elements were conducive to the propagation of the dominant bacteria, the secretion of enzymes, and the extraction rate of Cd in the soil. The possible enhancement mechanism of phytoremediation of cadmium by *A. pitti* combined with OG was that, on the one hand, APOG increased soil nutrient elements and enzyme activities promoted the growth of ryegrass. On the other hand, APOG activated Cd and boosted the movement of Cd from soil to ryegrass. This research offers insight for the combination of phosphate-solubilizing bacteria with an orange-peel-based activator to improve phytoremediation of Cd-contaminated soils and also provides a new way for the resource utilization of fruit residue.

## 1. Introduction

Cadmium is a highly toxic metal and is not part of the metabolic activities of animals and plants. With the advance of agricultural modernization, cadmium pollution in the soil of agricultural lands has been caused by the intensive use of chemical fertilizers and pesticides with an excessive content of toxic metals and by the excessive release of industrial effluents from mining and metallurgy [1]. China is a great agricultural country, and Hunan Province of China is a major rice growing area. Non-ferrous metal resources are rich, and farmland soil is seriously polluted by cadmium [2,3]. Cadmium in farmland soil will migrate to food and crops, and enter the food chain, endangering human health. When cadmium enters the human body, it destroys the normal physiological activities of the liver, kidney, and other organs of the human body, and then spreads to all parts of the human body along with the circulatory system, suppressing the activities of immune cells, and eventually evolves into cancer [4]. Therefore, how to handle the Cd pollution in soil economically and efficiently is a big puzzler that we have to face [5]. To remediate the Cd pollution in soil, several techniques have been proposed such as electrokinetic remediation, replacement of contaminated soil, chemical leaching, curing or stabilization, phytoremediation, etc. [6,7]. Compared with physicochemical remediation, phytoremediation has the characteristics of low cost, easy operation, excellent recovery without secondary pollution, and is being applied widely [8,9,10,11]. However, the efficiency of phytoremediation is often limited by the biomass and growth cycle of super-accumulated plants [12]. Ryegrass (*Lolium perenne L.*) has been widely used in the remediation of Cd-contaminated soil because of its fast growth rate and large biomass [13]. But its efficiency has been limited by the bioavailability of toxic metals. Therefore, activators have been used to increase the bioavailability of toxic metals and then enhance the effectiveness of the phytoremediation technique [14]. At present, ethylenediaminetetraacetic acid (EDTA), N, N′-1,2-ethanediylbis-1-Aspartic acid (EDDS), nitrilotriacetic acid (NTA), and glutamic acid, diacetic acid, tetrasodium salt (GLDA) have been used during the phytoremediation of toxic metals [15]. Traditional chelators may increase the risk of soil because it is hard to be biodegraded, resulting in potential ecological risks [16]. GLDA is a biodegradable and environmentally friendly chelating agent with a wide pH range [17], shown to significantly improve plant growth [18]. In addition, some research has shown that low molecular weight organic acids (LMWOA) contain acid radical ions, which are more easily combined with metal ions [19]. Cai et al. [20] showed that LMWOA could effectively promote the accumulation of Pb by *Centipedegrass* and enhance the biomass of *Centipedegrass.* It is worth noting that large amounts of LMWOA are found in fruit waste residues, and quantities of fruit residues are produced every year, such as bagasse, orange peel, watermelon peel, and so on [21]. If these fruit residues are used as an activator of LMWOA in toxic metals remediation, it will be a potential resource utilization method [22]. In consequence, developing an environmentally friendly activator by combining fruit residue extract and GLDA is really promising to promote the phytoremediation and the recycling of fruit residue. Ning et al. [23] made a composite activator with fruit residue extract, GLDA, and tea saponin, and found that it had enhanced effects on *Sedum alfredii* biomass and phytoremediation of Pb from soil, confirming that the compound activator derived from fruit residue is a potential phytoremediation enhancer.

As the most active component in soil, microorganisms also influence phytoremediation. With increasing research, microorganisms are being used as an accelerator during phytoremediation. Phosphate-solubilizing bacteria can dissolve phosphorus in the soil into available phosphorus [24]. Therefore, phosphate-solubilizing bacteria can be added to soil to enhance the amount of phosphorus that is accessible for plants, which will promote plant development [24]. Moreover, phosphate-solubilizing bacteria can change the morphology of Cd in soil through LMWOA production, extracellular enzymes, and so on, so as to improve Cd bioavailability and phytoremediation efficiency [25]. Jeong et al. [26] discovered that accumulation efficiency of cadmium in mustard vegetables tripled after the inoculation of phosphate-solubilizing bacteria due to the phosphorus-soluble capacity and the toxic metal activation of phosphate-solubilizing bacteria. At present, there are some studies on the enhancement of activators combined with microorganisms on phytoremediation. Usman and Mohamed [27] found that EDTA combined with arbuscular mycorrhizal fungi (AMF) enhanced Cd, Pb, Zn, and Cu concentrations in plants, and the effect was stronger than that of EDTA or AMF alone. However, the research about the enhancement of phytoremediation by combining activator and phosphate-solubilizing bacteria is few and the interaction mechanism is unclear.

This study explores a fresh concept for enhancement of phytoremediation in Cd-contaminated soil by combining activator and phosphate-solubilizing bacteria. The enhancement effect of phytoremediation of cadmium by phosphate-solubilizing bacteria (*Acinetobactre pitti*, *A. pitti*) combined with OG was studied and the enhancement mechanism of phytoremediation of cadmium by *A. pitti* combined with OG was proposed in a controlled experiment. The specific contents of the research are as follows: (1) The phytoremediation of cadmium from soil to ryegrass. (2) The enzyme activities and diversity of bacterial communities in soil. (3) Relationships of nutrient elements with bacterial community, enzyme activity, and plant extraction efficiency.

## 2. Materials and Methods

### 2.1. Orange Peel Residue-Based Activator

Hunan province is the main production area for oranges, where orange residue is readily available, previous research results showed that, a 1:1 (*v*/*v*) ratio of orange peel residue extract to GLDA was adequate to prepare an orange peel residue-based activator (OG). Orange peel residue waste obtained from the local fruit market was cleaned with ultrapure water 2–3 times and wiped dry with absorbent paper. The orange residue was broken up in a blender after being mixed with ultrapure water in a proportion of 1:5 (*w*/*w*). The mixture was then centrifuged at 5000 r min^−1^ for ten minutes and the supernatant isolated. GLDA (Nouryon, Shandong, China) was diluted to a concentration of 1.6% (*v*/*v*) and mixed with fruit residue extract at a ratio of 1:1 (*v*/*v*), which constituted the OG [28].

### 2.2. Phosphate-Solubilizing Bacteria

#### 2.2.1. Cultivation of Phosphate-Solubilizing Bacteria

*Acinetobactre pitti* (*A. pitti*, one kind of phosphate-solubilizing bacteria, deposit number is CICC10526) was bought from China Center of Industrial Culture Collection (CCICC). According to the operation manual, *A. pitti* was cultured with beef extract peptone medium: 5.0 g peptone, 3.0 g beef extract, 5.0 g NaCl, 1 L ultrapure water, pH = 7 (2% agar was added for solid medium). The freeze-dried bacteria powder stored in ampoule was dissolved in a small amount of liquid beef extract peptone medium, and the fully dissolved bacteria suspension was transferred to liquid beef extract peptone medium, and placed in a constant temperature incubator at 30 °C for 48 h to resurrect. The resurrected bacteria were inoculated on fresh beef extract peptone slant culture medium and incubated in 30 °C for 24 h. Then, the bacteria on slant culture medium were transferred into sterile water to prepare bacterial suspension with a concentration of 1 × 10^8^ CFU mL^−1^, which was inoculated into liquid beef extract peptone medium and shaken at 150 r min^−1^ and 30 °C for 24 h with a logarithmic growth period. This is the bacterial inoculum solution for the pot experiments.

#### 2.2.2. The Pro-Growth Secretions of Phosphate-Solubilizing Bacteria

Chrome azurol sulphonate (CAS) medium: each 100 mL contained 20% sucrose solution of 1 mL, 10% acid hydrolyzed casein (3 mL), 1 mmol L^−1^ CaCl_2_ (100 μL), l mmol L^−1^ MgSO_4_·7 H_2_O_2_. Phosphate buffer and CAS dye solution (0.06 g L^−1^ CAS solution) were slowly added, along with 0.0027 g L^−1^ FeCl_3_ · 6H_2_O, 0.073 g L^−1^ hexadecyl trimethyl ammonium bromide solution): 5 mL each at about 60 °C. The bacterial suspension of *A. pitti* (1 × 10^8^ CFU mL^−1^) was inoculated into the chrome azurol sulphonate (CAS) medium at a concentration of 2% and cultured at 150 r min^−1^ at 30 °C for 3 days. Bacteria were removed from the fermentation liquid, and the supernatant was subject to the CAS blue liquid detection method; the absorption value of siderophore was determined by a visible light spectrophotometer [29,30].

The IAA solid (Coolaber, Beijing, China) was weighed, dissolved in a small amount of anhydrous ethanol, and then ultrapure water was added to prepare a working liquid with a concentration of 100 mg L^−1^. The working liquid was diluted to a standard sample with a series concentration of 0, 10, 20, 40, 60, 80, 100 mg L^−1^. The standard curve was drawn by adding the concentration of IAA as the horizontal coordinate and OD value as the vertical coordinate. *A. pitti* was inoculated into Luria–Bertani (LB) medium containing 200 mg L^−1^ tryptophan (2% concentration), cultured at 30 °C for 3 days. The fermentation solution was centrifuged at 6000 r min^−1^ for 15 min, and the supernatant was added to a Salkowski reactant (1:1, *v*/*v*). The reaction was kept at room temperature and in the dark for 30 min. The absorbance was measured at 530 nm and the IAA content was calculated according to the standard curve [31].

The bacterial suspension of *A. pitti* was inoculated into beef extract peptone medium at a 2% inoculum rate, cultured at 150 r min^−1^ at 30 °C for 3 days, and then the fermentation liquid was centrifuged at 8000 r min^−1^ for 10 min, and the supernatant was taken for testing. 1-aminocyclopropane-1-carboxylate (ACC) deaminase was detected by an ACCdase ELISA Kit (ZCIBIO, Shanghai, China). The detection principle of the kit was as follows: the target antibody was coated in a 96-well microplate to make a solid phase carrier. Standard substances or samples were added into the micropores, where the target substance bound to the antibody attached to the solid phase carrier. Then, a horseradish-peroxidase-labeled antibody was added, the unbound antibody was washed and thoroughly washed again, and 3,3′,5,5′-Tetramethylbenzidine (TMB) was added for color development. TMB was catalyzed by peroxidase to turn blue and converted to the final yellow color by the action of acid. The depth of the color was positively correlated with the concentration of the target in the sample. Determination of absorbance (OD) at 450 nm was performed by a microplate reader.

### 2.3. Pot Experiment

Cadmium-polluted agricultural soil was obtained from Jiaoxi Village, Liuyang City, Hunan Province, China. The depth of soil sample collection was 0~20 cm. Ten sample areas were designed, and each sample area was collected within the range of 5 m × 5 m by the plum blossom sampling method. The soil was sieved (10-mesh screen) after natural drying and grinding. The properties of experimental soil were listed in Table 1.

A sample of 250 g soil was put in an 11 cm diameter plastic pot. The treatment was added to 80 mL ultrapure water without OG and *A. pitti*, namely CK. Then, the treatment was added to 20 mL OG, and 60 mL ultrapure water to soil, namely OG. Finally, the treatment was added to 20 mL OG and 60 mL ultrapure water and *A. pitti* to be inoculated after germination, namely APOG. Three treatments were set up with 5 parallel samples for a total of 15 samples. An amount of 20 ryegrass seeds were evenly sown in each group, in which the seeds were steeped in ultrapure water for another 24 h after being disinfected in 1% sodium hypochlorite solution for 15 min. The pot experiment was carried out at 25–30 °C. After seed germination, the pots were weighed every two days and supplemented with pure water so that the soil moisture content was 60~65% of the maximum field capacity. Ten days after seed germination, 5 inoculation sites were set in the pot, with an inoculation depth of 3 cm below the soil surface, and 3 mL of bacterial solution was inoculated at each site. On the 30th day after germination, the above-ground part of the ryegrass was harvested to detect Cd, the height and fresh weight. An amount of 45 g of soil was collected from the rhizosphere area of the ryegrass, and the sampling depth was 0~5 cm. The soil was dried and ground to analyze the nutrients, total cadmium, available cadmium, and enzymatic activity. An amount of 10 g of soil was frozen in a −20 °C refrigerator for subsequent microbial diversity analysis.

### 2.4. Analysis of Plant Samples

The plants’ heights were measured with a ruler with a precision of 1 mm and their fresh weights were weighed and recorded using a ten-thousandth electronic scale (Model number: FA2004, Shanghai Sunny Hengping Scientific Instrument Co., Ltd., Shanghai, China). Ryegrass samples were desiccated for 10 min at 110 °C and dried at 70 °C to maintain a constant mass. Ryegrass samples were ground and sieved through a 10-mesh sieve, and 0.1 g were weighed and digested with nitric acid. The content of Cd in ryegrass was determined by inductively coupled plasma mass spectrometry (ICP-MS, PerkinElmer NexION 350, America, the detection limit was 0.002 μg L^−1^).

### 2.5. Analysis of Soil Samples

#### 2.5.1. Nutrient Elements

The technique described by Xiao et al. [32] was used to analyze the soil nutrient elements. The total nitrogen (TN) of the soil was measured by the Kjeldahl method. The total phosphorus (TP) and available phosphorus (AvP) of the soil were determined by the molybdenum-antimony anti-spectrophotometric method. The alkaline hydrolysis nitrogen (AN) of soil was detected by alkaline hydrolysis diffusion method. The available kalium (AK) in the soil was determined by ammonium acetate flame spectrophotometry, and total organic matter (TOM) was determined by chromate redox titration.

#### 2.5.2. Toxic Metals Elements Detection

An amount of 0.1 g dried pot soil passed through a 100-mesh sieve and was weighed and digested by HNO_3_-HF-HClO_4_, and then the total Cd in the digestive liquid was determined by ICP-MS [33]. Then, an amount of 2 g of dried pot soil passed through 20-mesh sieve and was extracted by 20 mL 0.01 mol L^−1^ CaCl_2_, and then the available Cd was determined by ICP-MS. Blank and parallel samples were set up in the detection process, and the national standard soil sample GBW07402 (Institute of Geophysical and Geochemical Exploration, Chinese Academy of Geological Sciences, Beijing, China) was used for quality control; the recovery was higher than 90%.

#### 2.5.3. Soil Enzyme Activity

The activities of phosphatase, sucrase, catalase, and urease were determined by phenyldisodium phosphate colorimetric method, 3,5-dinitrosalicylic acid colorimetric method, potassium permanganate titration method, and sodium phenol-sodium hypochlorite colorimetric method, respectively [34].

### 2.6. DNA Extraction, PCR Amplification, and High-Throughput Sequencing

A PowerSoil^®^ DNA Isolation Kit (ANBIOSCI TECH LTD, China) was used to extract the soil microbial genome DNA from 0.25 g of freeze-dried pot soil samples according to the manufacturer’s instructions.

PCR amplification was performed using bacterial 16S rRNA primers 27F (AGRGTTTGATYNTGGCTCAG) and 1492R (TASGGHTACCTTGTTASGACTT), and PCR products were recovered and purified. After purification, qualified PCR products were constructed to form a sequencing library (SMRT Bell) and sequenced using PacBio Sequel. The raw sequencing data were deposited into the NCBI Sequence Read Archive (SRA) database (PRJNA938747, https://dataview.ncbi.nlm.nih.gov/object/PRJNA938747?reviewer=f24c1p94sq5ikb6p07b63tae5b, accessed on 31 July 2023).

### 2.7. Data Analysis

The percentage of the difference of Cd in the soil before and after phytoremediation to the total amount of Cd before phytoremediation was calculated in a decrease ratio of Cd (MDR). The ability of the plants to extract Cd from the soil was indicated by the bioconcentration factor (BCF). The calculation formulas are as follows:

(1) $$MDR=\frac{C_{0}-C_{m}}{C_{0}}\times 100\%$$

In which:

C_0_—the content of total Cd in soil before phytoremediation, mg kg^−1^

C_m_—the content of total Cd in soil after phytoremediation, mg kg^−1^

(2) $$BCF=\frac{C_{p}}{C_{0}}\times 100\%$$

In which:

C_p_—the content of Cd in the plant, mg kg^−1^

Three parallel samples were tested for each indicator, and the final test result was represented by mean ± standard deviation. IBM SPSS Statistics (Version 25.0, Armonk, NY, USA: IBM Corp.) was used for statistical analysis of the experimental data, and the significant differences were analyzed by LSD (least significant difference) method at the level of *p* < 0.05. RDA analysis was performed by Canoco (Version 5, Microcomputer Power, Ithaca, NY, USA).

## 3. Results and Discussion

### 3.1. Growth Promoting Properties of A. pitti 

In the rhizosphere soil region of plants, there are abundant microbial communities, and rhizosphere microorganisms are the most active components of rhizosphere soil, which form a complex plant–soil–microbial system with plants and soil. Microorganisms secrete siderophore, IAA, ACC deaminase, and other substances to regulate plant growth [35]. Siderophore can promote the absorption of Fe element by plants, IAA is a hormone that promotes plant growth, and ACC deaminase can degrade ACC, which is the precursor substance of ethylene synthesis, so as to reduce the level of ethylene and reduce the inhibition of excessive ethylene on the growth of plant roots and stems [36]. These substances that promote growth are conducive to the growth of plants, increasing the biomass of plants. However, plants with good growth conditions and strong stress resistance will have a larger biomass, which is conducive to the absorption of more toxic metals, so as to achieve the purpose of enhancing the efficiency of phytoremediation [37]. Therefore, the growth-promoting properties of phosphate-solubilizing bacteria were studied by detecting the siderophore, IAA, and ACC deaminase during the pure culture experiment. The content of siderophores produced by *A. pitti* reached 15.95%. The yield of IAA was 25.75 mg L^−1^, 5.01 times that without *A. pitti.* The production of ACC deaminase was 2.97 ng mL ^−1^, 1.46 times that without *A. pitti.* The results showed that *A. pitti* had the ability to produce growth-promoting substances, which was consistent with the characteristics of phosphate-solubilizing bacteria [38]. The strains with these abilities can promote plant growth, increase the content of chlorophyll and soluble sugar in plant leaves, and reduce the content of malondialdehyde in plants. These growth-promoting effects can indirectly improve the accumulation of toxic metals in plants and also increase the efficiency of phytoremediation [35,39].

### 3.2. Effect of APOG on the Phytoremediation of Cadmium from Soil to Ryegrass

**Figure 1:**
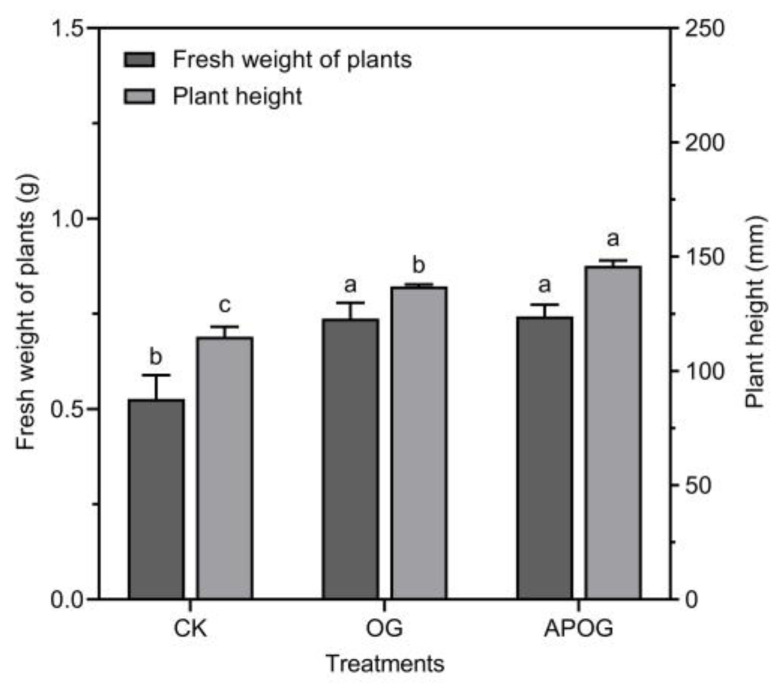
Height and fresh weight of ryegrass in different treatments. Letter labels (a, b, c) were used to indicate significant differences between the groups. The statistical analysis method was LSD at the level of *p* < 0.05.

During the phytoremediation of toxic metals, the biomass of the plant was extremely important to the efficiency of the extraction; in general, plants with higher biomass are more likely to absorb more toxic metals [12]. The fresh weight and plant height were detected to evaluate the status of plant growth in Figure 1. The fresh weight of ryegrass in OG and APOG were about 1.4 times that of CK, which were 0.7400 g and 0.7433 g, respectively. The average height of ryegrass in CK, OG, and APOG were 115, 132, and 146 mm. Compared with CK, the height of ryegrass in OG and APOG significantly increased by 14.78% and 21.23%. In summary, the growth-promoting effect of APOG on ryegrass was greater than that of OG. This was due to the fact that adding *A. pitti* enhanced the amounts of nutrient elements (the data of TN, TP, AvP, AN, AK, and TOM were shown in Supplementary Materials Figure S1), which was conducive to the growth of ryegrass. Meanwhile, the interaction between microorganisms and plant roots could improve the utilization rate of nutrients, accelerate plants growth, and inhibit disease [40,41].

**Figure 2:**
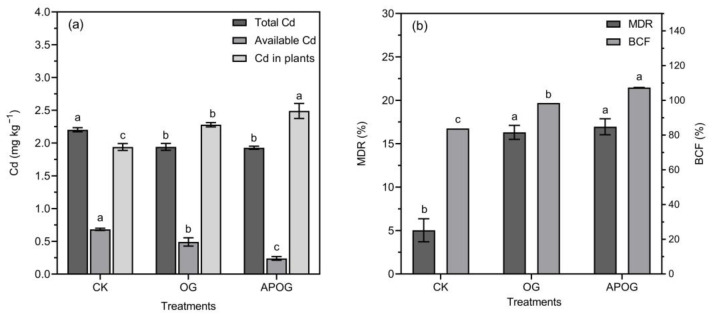
The contents of total and available cadmium in soil and cadmium in plants (**a**), the decrease ratio of Cd (MDR), the bioconcentration factor (BCF) (**b**) in different treatments. Letter labels (a, b, c) were used to indicate significant differences between the groups. The statistical analysis method was LSD at the level of *p* < 0.05.

Studying the amounts of total Cd and available Cd in soil, as well as its content in plants, could reflect the migration and conversion of Cd in the soil–plant system, the results of which are shown in Figure 2a. Compared with CK, the content of Cd in ryegrass increased by 1.18 times and 1.28 times in OG and APOG. With the addition of OG and APOG, the total Cd and available Cd showed a decreasing trend, in which the content of available Cd in the CK group was 38.89% higher than that in OG group, and 184.19% higher than that in the APOG group. This was because both *A. pitti* and OG activated Cd, and the available Cd was absorbed and utilized by ryegrass, resulting in the decrease of total Cd and available Cd in soil, and the increase of Cd in ryegrass.

The MDR and BCF were computed in order to assess the phytoremediation ability of ryegrass, as shown in Figure 2b. The variation trend of MDR and BCF in different treatments was APOG > OG > CK. Compared with CK, the MDR was 3.24 times in the OG group and 3.37 times in the APOG group. Meanwhile, BCF was 1.18 times in the OG group and 1.28 times in the APOG group, higher than that in CK group, respectively. The MDR and BCF were improved significantly by adding OG and APOG, indicating that OG and APOG facilitated the transport of Cd from soil to plants. The effect of APOG was higher than that of OG. On the one hand, LMWOA secreted by *A. pitti* in APOG further enhanced the activation of Cd by OG [42]; on the other hand, APOG could enrich the soil nutrients that were conducive to the growth of ryegrass and then increase the biomass of ryegrass, so as to strengthen ryegrass’ ability to extract Cd [43].

### 3.3. Effect of APOG on the Enzyme Activities in Soil

The transformation of soil nutrients is usually related to soil enzyme activities, and the soil enzymes can reflect the substance circulation and energy conversion. For example, the activity of urease can reflect the conversion of organic nitrogen in soil, the activity of catalase can reflect the conversion rate of soil organic matter, and the soil phosphatase activity reflects soil P level to a certain extent [44]. And the increase of nutrient elements and enzyme activity is beneficial to plant growth [45].

**Figure 3:**
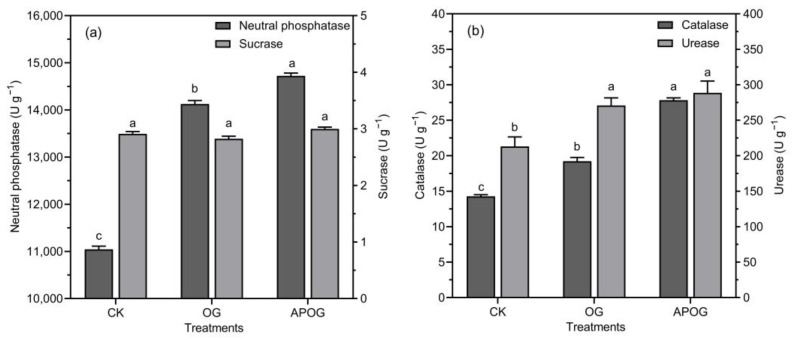
Contents of neutral phosphatase and sucrase (**a**), catalase and urease (**b**) in different treatments. Letter labels (a, b, c) were used to indicate significant differences between the groups. The statistical analysis method was LSD at the level of *p* < 0.05.

The changes of the activities of neutral phosphatase, sucrase, catalase, and urease in different treatments were investigated in this study. Figure 3a showed that neutral phosphatase activity from more frequent to less frequent was APOG, OG, and CK. APOG was 1.33 times that of CK, which may be because *A. pitti* could secret phosphatase [46]. The activities of catalase and urease were presented in descending order as APOG > OG > CK in Figure 3b. The catalase activity in OG and APOG was 1.35 and 1.95 times higher than that in CK. In general, catalase activity would decrease under toxic metals stress [47]. The data show that the catalase activity in APOG group is much higher than that in the other two groups. This is because the addition of APOG promoted the transfer of Cd from soil to ryegrass and reduced the content of Cd in soil, thus alleviating the stress of catalase caused by Cd. Urease activity increased by 27.13% and 35.48% in OG and APOG than that in CK, respectively. And it is conducive to the conversion of organic nitrogen to available nitrogen, which is similar to the change of AN in this study.

### 3.4. Effect of APOG on the Diversity of Soil Bacterial Communities

In this study, a total of 116873 CCS sequences were obtained from nine samples, in which the number of effective sequences for subsequent analysis after removal of chimeras was 104,226, with an average length about 1437 bp. The operational taxonomic units (OTU) dilution curve in the samples tended to be flat, indicating that species in this environment did not increase significantly with the increasing of sequencing quantity. The sequencing quantity was sufficient to truly reflect the composition of soil bacterial community after the addition of OG and APOG and could be used for subsequent data analysis. 

The Shannon index, Simpson index, and Ace index were used to describe the diversity and richness of soil bacteria OTUs [48]. The Shannon index and Simpson index were directly proportional to the diversity of the bacterial community [48]. The Ace index was used to evaluate the richness of species composition in the sample. The greater the ACE index was, the greater the richness of species in the community. As shown in Table 2, the Shannon index, Simpson index, and Ace index are the highest in the OG group, indicating that the bacterial diversity and richness in the OG group were the highest. However, the Shannon index and Simpson index show that there is no significant difference in bacterial diversity between the CK group and the APOG group, but the Ace index of AOPG is greater than CK. This is because OG contains fruit residue, and there are many LMWOAs, sugars, cellulose, and other nutrients in fruit residue, which is an ideal carbon source for soil microorganisms because it intensifies the activities of soil microorganisms and improves the diversity of the microbial community [49,50,51]. However, the addition of *A. pitti* competes with the indigenous microorganisms in the soil to some extent, and the intraspecific struggle leads to the bacterial diversity of the APOG group being lower than the OG group, and this is consistent with the findings of Jeong et al. [27].

**Figure 4:**
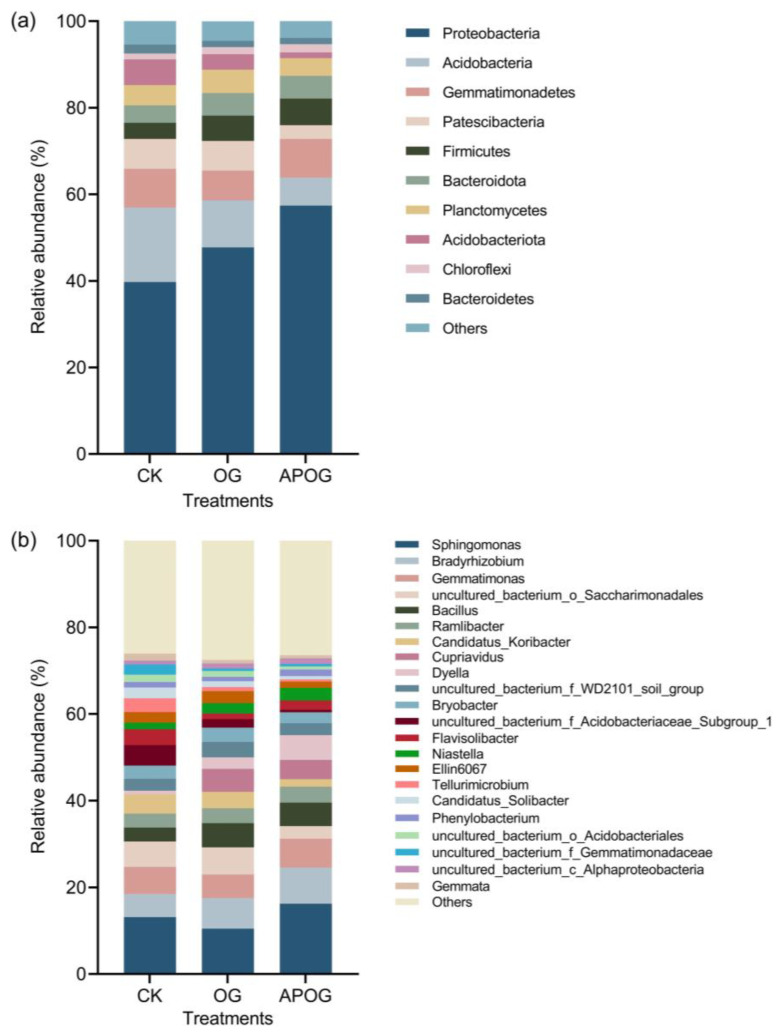
Relative bacterial abundance at phylum level (**a**) and genus level (**b**) in different treatments.

Figure 4 shows the relative abundance of bacteria at phylum level and genus level in soil in different groups. As shown in Figure 4a, bacteria in the soil were mainly distributed in 10 phyla at the phylum level, in which the relative abundance of *Proteobacteria* was the highest, accounting for 39.74–57.38% of the total number of bacteria, and it was in a descending order with APOG, OG, and CK. *Acidobacteria* was the second largest, accounting for 6.5–17.24% of the total number of bacteria. *Proteobacteria* and *Acidobacteria* were the main microorganisms, with a large proportion in the Cd-contaminated soil at phylum level; this was consistent with the findings of many researchers [52]. This is because *Proteobacteria* and *Acidobacteria* have strong adaptability to extreme environments [53]. And *Acidobacteria* were one kind of important bacteria in soil and can degrade plant residues and participate in carbon metabolism, photosynthesis, and other material cycle and ecological environment construction [54]. In addition, the relative abundances of *Gemmatimonadetes*, *Patescibacteria,* and *Firmicutes* were also high, accounting for 6.83–8.94%, 3.17–6.9%, and 3.73–6.17%, respectively. The relative abundance of the three groups was different and varied greatly. At the same time, there were also great differences in the relative abundance of other bacteria. This may be due to the ability of different types of bacteria to adapt to the environment.

Figure 4b shows the proportion of bacteria with an abundance greater than 1% in the soil at the genus level. *Sphingomonas* had the highest abundance in the three groups, which accounted for 13.15%, 10.46%, and 16.22% in CK, OG, and APOG, respectively. This is because *Sphingomonas* has the characteristics of a fast reproduction speed and a strong growth adaptability to toxic metal pollution in the environment [55]. Moreover, *Sphingomonas* was a part of *Proteobacteria* organisms, and *Sphingomonas* has the ability to remove refractory pollutants [56]. In this research, the combination of *A. pitti* and OG significantly promoted the growth of *Sphingomonas*. The second most abundant genus was *Bradyrhizobium*, which accounted for 5.31%, 7.08%, and 8.31% in CK, OG, and APOG, respectively. Furthermore, the number of *Bradyrhizobium* increased significantly after inoculating *A. pitti*, and studies have shown that *Bradyrhizobium* can promote the phytoremediation of toxic metals [57]. In addition, *Bacillus*, *Gemmatimonas*, *Ramlibacter*, and *Candidatus-Koribacter* were the top 10 bacterial genera, with an abundance over 1%.

### 3.5. Relationships of Nutrient Elements with Bacterial Community, Enzyme Activity, and Plant Extraction Efficiency

**Figure 5:**
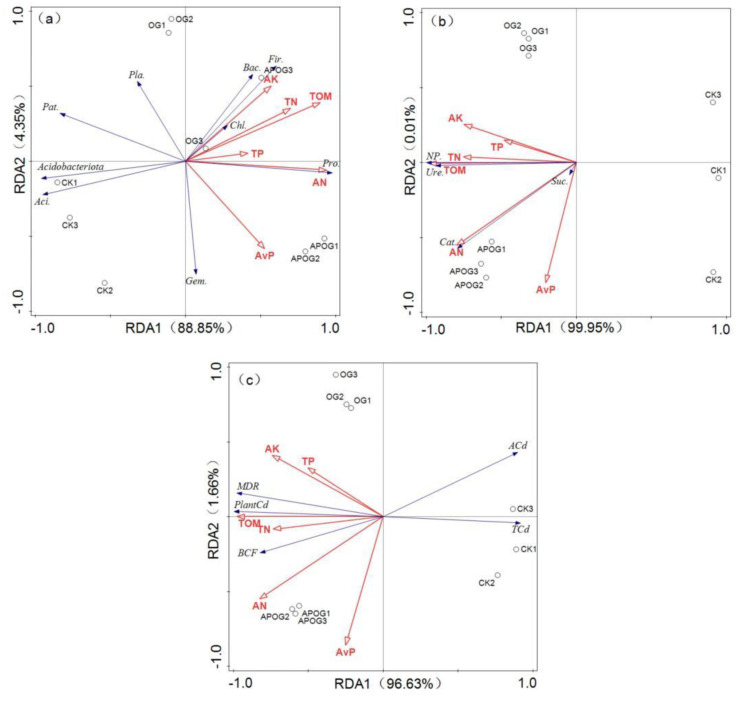
RDA analysis of nutrient elements with bacterial community (**a**), nutrient elements with enzyme activity (**b**), nutrient elements with Cd transformation (**c**). (Pro.—*Proteobacteria*, Aci.—*Acidobacteria*, Gem.—*Gemmatimonadetes*, Pat.—*Patescibacteria*, Fir.—*Firmicutes*, Bac.—*Bacteroidota*, Pla.—*Planctomycetes*, Chl.—*Chloroflexi*, NP.—Neutral Phosphatase, Suc.—Sucrase, Cat.—Catalase, Ure.—Urease, ACd—Available Cd, TCd—Total Cd, Plant Cd—Cd in plant).

The relationships of the soil bacterial community structure, enzyme activity, total and available cadmium levels in soil, cadmium in ryegrass, MDR, and BCF with nutrient elements in the three groups were analyzed by RDA analysis and shown in Figure 5.

Figure 5a showed the RDA analysis of nutrient elements and soil bacterial community structure. The two ordination axes explained 93.20% of the difference in soil bacterial community composition, and the contribution rate of the first ordination axis was 88.85%. It reflected that the composition of soil bacterial community was affected dominantly by AN (F = 24.7, *p* < 0.05) and TP (F = 1.4, *p* < 0.05), and the explanation rates were 67.1% and 16.6%, respectively. RDA analysis of soil neutral phosphatase, sucrase, catalase, and urease activity with nutrient elements is shown in Figure 5b. The first and second ranking axes explained 99.96% of the four kinds of enzymes. TOM was the main influencing factors for the enzymes activities (F = 195, *p* < 0.05) and the explanation rate was 96.5%. Figure 5c reflected the RDA analysis results of MDR, BCF, Cd content in plants, and total and available cadmium content in soil with nutrient elements. The contribution rates of the first and second ordination axis were 96.63% and 1.66%, respectively. The main influencing factors were TOM (F = 81.7, *p* < 0.05) and AK (F = 3.1, *p* < 0.05), and the explanation rates were 92.1% and 3.1%, respectively.

Studies have shown that habitats with high organic carbon, nitrogen, and phosphorus content are more suitable for the growth of soil bacteria and the increase of soil bacteria diversity [58]. In this research, AK, TN, TOM, TP, and AN were positively correlated with the abundance of *Bacteroidota*, *Firmicutes*, *Chloroflexi*, *Proteobacteria*, and negatively correlated with the abundance of *Patescibacteria*, *Acidobacteriota*, *Acidobacteria*. AvP was positively correlated with the richness of *Proteobacteria* and *Gemmatimonadetes*. This may be because *Chloroflexi* could improve the circulation of C and N and improve soil nutrients [59]. Moreover, the increase of nutrient elements was conducive to plant growth, thus enhancing plant extraction of Cd from soil and reducing the inhibition of Cd in soil by *Proteobacteria* [60]. But AK, TN, TOM, TP, and AN were negatively correlated with the richness of *Acidobacteria*, which may be because *Acidobacteria* is more suitable for growth under poor nutrient conditions [61]. Additionally, nutrient elements were positively correlated with the activities of neutral phosphatase, sucrase, catalase, and urease. Soil enzyme activity could accelerate soil metabolism and promote nutrient circulation, which was conducive to plant growth [62]. Next, AK, TP, AN, TOM, AvP, and TN were positively correlated with the MDR, BCF, and Cd in plants and all nutrient elements were negatively correlated with available Cd and total Cd in soil. This was because the orange-residue-based activator and *A. pitti* enhanced the Cd bioavailability as well as promoted the transfer of Cd from soil to ryegrass, resulting in the reduction of available Cd and total Cd and the enhancement of phytoremediation efficiency. The relationship of nutrient elements with bacterial community, soil enzyme, and plant extraction efficiency provides the basis for the enhancement mechanism of phytoremediation of Cd by *A. pitti* combined with OG.

### 3.6. Enhancement Mechanism of Phytoremediation of Cadmium by A. pitti Combined with OG

**Figure 6:**
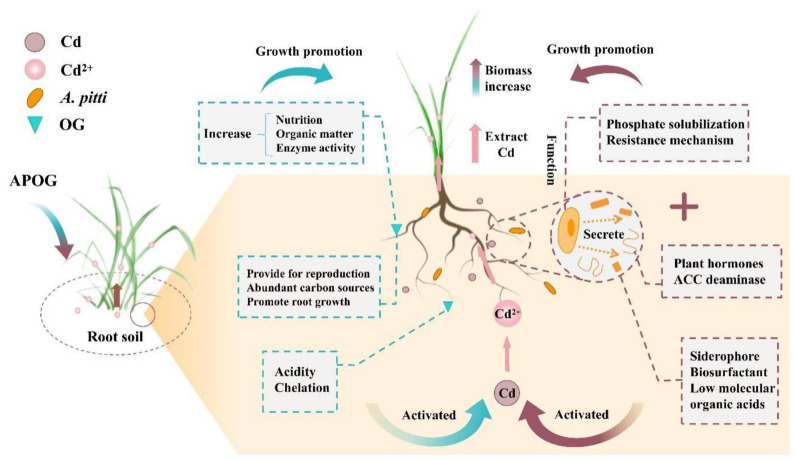
Enhancement mechanism of phytoremediation of Cd by phosphate-solubilizing bacteria combined with the orange-residue-based activator.

Based on the relationships of nutrient elements with bacterial community, enzyme activity, and plant extraction efficiency, the enhancement mechanism of phytoremediation of cadmium by *A. pitti* combined with OG is summarized as shown in Figure 6.

The raw material of OG contains abundant LMWOA, which may reduce the pH of soil, and then the insoluble heavy metals will be activated more easily and converted into bioavailable forms [63,64]. Meanwhile, the orange-residue-based activator could improve plant growth and increase plant biomass by increasing soil nutrients, organic matter, and enzyme activity [65,66]. It was also important that the rich sugars and cellulose in fruit residue can provide a carbon source for microorganisms in soil [49,50]. This provided the basis for enhancing the abundance and diversity of soil bacteria and provided the possibility for the combination of OG and *A. pitti*.

In the process of plant growth, the root environment is closely related to phosphorus-solubilizing bacteria, and most of them have a mutually beneficial and symbiotic relationship [67]. Studies have shown that phosphate-solubilizing bacteria as plant-growth-promoting rhizobacteria (PGPR), which could stimulate plant root exudates, induce the plant resistance’s system and produce antibiotics to inhibit disease, and root exudates provide a carbon source and energy for phosphate-solubilizing bacteria to a certain extent [68]. In this study, the biomass of ryegrass increased significantly, and the activities of neutral phosphatase, catalase, and urease were significantly improved after the inoculating *A. pitti* treatment. This indicated that the metabolic activity of C, N, and P in the root environment was enhanced, which was conducive to plant growth and the generation of a large amount of root exudates, thus increasing the bioavailability of cadmium and the efficiency of plant extraction.

In this work, phosphate-solubilizing bacteria are also crucial. On the one hand, *A. pitti* exerts its growth-promoting effect on plants by producing siderophores, IAA, ACC deaminase, and other substances. IAA enhanced the development of plant roots, and ACC deaminase could inhibit the synthesis of ethylene and relieve external stress on plants [69,70]. Siderophores could provide nutrient transport for plants under a toxic metals stress environment, so as to alleviate the toxicity of heavy metals to plants [71]. Moreover, in a harsh environment such as one polluted by diseases, insects, and toxic metals, phosphate-solubilizing bacteria could produce antibiotics, hydrogen cyanide, and other substances to promote the plant’s stress resistance [72]. Separately, phosphate-solubilizing bacteria could transform soil phosphorus into available phosphorus through acid dissolution and other functions, thus providing available phosphorus for plant growth [73]. These effects provided the basis for the healthy growth of ryegrass in a Cd-polluted environment and the increase of ryegrass biomass. On the other hand, phosphate-solubilizing bacteria could activate Cd by secreting LMWOA, biosurfactants, and siderophores [74,75]. Many studies showed that LMWOA could enhance the mobility of toxic metals by complexing, biosurfactants could dissociate metal ions from soil particles, and siderophores could promote the transportation of toxic metals by forming metal-siderophore chelates [19,74,76]. These effects increased the bioavailability of Cd and made it more easily absorbed by ryegrass.

In summary, the combination of *A. pitti* and OG enhanced the phytoremediation of Cd by ryegrass through two possible mechanisms in this research. Firstly, *A. pitti* and OG increased the availability of Cd, and then promoted the transfer of Cd from soil to ryegrass. Secondly, *A. pitti* and OG increase the biomass of ryegrass and enhance its tolerance to Cd stress. Studies have found that in EDDS-assisted extraction of Cu by alfalfa, inoculation of *Paenibacillus mucilaginosus* and *Sinorhizobium meliloti* could reduce the toxicity of metals to plants and enhance phytoremediation [77], which is similar to the results of this study. Therefore, these mechanisms suggested that the combination of phosphate-solubilizing bacteria and the orange-residue-based activator had a certain potential to improve the efficiency of phytoremediation of Cd by ryegrass.

## 4. Conclusions

In this study, the enhancement effect of orange-residue-based activator combined with phosphate-solubilizing bacteria on soil Cd repair of ryegrass was discussed. The results showed that phosphate-solubilizing bacteria combined with orange-residue-based activator could improve soil nutrients and increase soil enzyme activity and bacterial diversity, which was conducive to the increase of beneficial bacteria, thus increasing the biomass of ryegrass. In addition, phosphate-solubilizing bacteria and the orange-residue-based activator significantly activated Cd and increased the concentration of available Cd in soil. These combined effects are conducive to the absorption of Cd in soil by ryegrass and the remediation of Cd soil pollution.

## Figures and Tables

**Table 1:** Soil physicochemical properties.

Indicators	Determined Value	Indicators	Determined Value
pH	4.57 ± 0.80	Available phosphorus (mg kg^−1^)	0.63 ± 0.017
EC (μS cm^−1^)	122.50 ± 2.10	Available potassium (mg kg^−1^)	149.06 ± 7.33
Total nitrogen (mg g^−1^)	3.27 ± 0.063	Total organic matter (%)	1.66 ± 0.03
Total phosphorus (mg g^−1^)	0.70 ± 0.014	Total cadmium (mg kg^−1^)	2.32 ± 0.06
Alkaline hydrolysis nitrogen (mg g^−1^)	0.36 ± 0.079	Available cadmium (mg kg^−1^)	0.35 ± 0.01

**Table 2:** Diversity index of the bacterial community in soil in different treatments.

Sample	Shannon	Simpson	Ace
CK	6.76 ± 0.083	0.97 ± 0.004	507.48 ± 10.217
OG	6.80 ± 0.156	0.98 ± 0.001	542.22 ± 47.005
APOG	6.50 ± 0.057	0.97 ± 0.003	524.38 ± 7.536

## Data Availability

Data will be made available on request.

## Supplementary Materials

The following supporting information can be downloaded at: https://www.mdpi.com/article/10.3390/plants12142727/s1, Figure S1: Changes of nutrient elements in different treatments.
