# Supplementary figures and images for: Effect of the Combination of Phosphate-Solubilizing Bacteria with Orange Residue-Based Activator on the Phytoremediation of Cadmium by Ryegrass
Source: Plants (Basel). 2023 Jul 22;12(14):2727. doi: 10.3390/plants12142727 (PMC10384834; doi:10.3390/plants12142727)

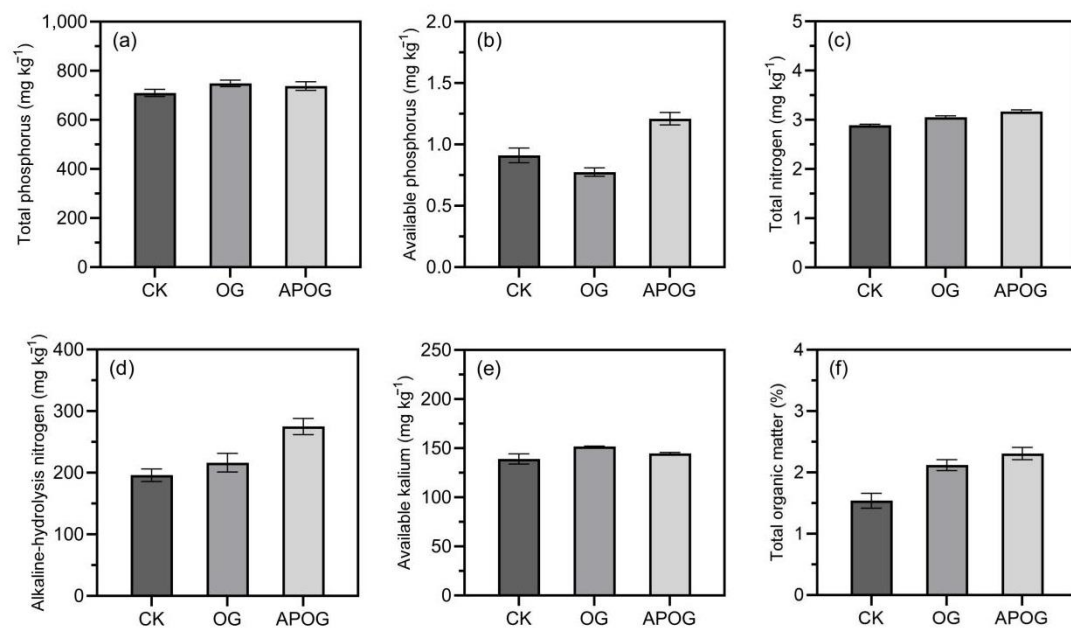

**Figure S1.** Changes of nutrient elements in different treatments.

Supplement: Supplementary file 1 [file plants-12-02727-s001.zip › plants-2447757-supplementary.pdf]
